# Social and environmental determinants of child health in Mongolia across years of rapid economic growth: 2000-2010

**DOI:** 10.1186/s12939-017-0684-x

**Published:** 2017-10-30

**Authors:** Nehal Joshi, Bolormaa Bolorhon, Indermohan Narula, Shihua Zhu, Semira Manaseki-Hollan

**Affiliations:** 1 Medicine, University of Birmingham, Birmingham, UK; 2 Medical Student, Health Sciences University of Mongolia, Ulaanbaatar, Mongolia; 3 Team Leader, Global Fund LFA, Ulaanbaatar, Mongolia; 4 Research Fellow in Health Economics and Mathematical Modelling, Public Health, Epidemiology and Biostatistics, School of Health and Population Sciences, College of Medical and Dental Sciences, University of Birmingham, Birmingham, UK; 5 Clinical Senior Lecturer, Department of Public Health, Epidemiology and Biostatistics, School of Health and Population Sciences, College of Medical and Dental Sciences, University of Birmingham, Birmingham, UK

**Keywords:** Health equity, Child nutrition, Maternal education, Socioeconomic status, Immunisation coverage, Multiple indicator cluster surveys (MICS), Mongolia

## Abstract

**Background:**

To understand the effect of economic growth on health, we investigated the trend in socio-economic and regional determinants of child health in Mongolia. This Central Asian country had the fastest economic growth amongst low and middle-income countries (LMICs) from 2000 to 2010 and a healthcare system in transition.

**Methods:**

Data was from Mongolian multiple indicator cluster surveys (MICS) in 2000, 2005 and 2010. Child nutrition/growth was measured by height-for-age z-score (HAZ), weight-for-age z-score (WAZ), prevalence of stunted (HAZ < −2) and underweight (WAZ < −2) children. Access to health care was measured by prevalence of fully immunised children <5 years. Multivariate multi-level logistic mixed modelling was used to estimate the effect of socio-economic and environmental health determinants on each outcome in each year; 2000, 2005 and 2010. T-tests were used to measure significant change in HAZ and WAZ over the decade.

**Results:**

Overall, from 2000 to 2010, there was a significant improvement (*p* < 0.001) in all three outcomes, but the effect of socio-economic factors increased on both stunting and weight. In 2000, region was a significant determinant: children living in three provinces were significantly more likely to be stunted and less likely to be immunised than Ulaanbaatar, but this was not significant by 2010. By 2010, none of the factors were significant determinants of immunisation in children. In 2000, economic status had no effect on stunting (OR = 0.91; 95%CI:0.49,1.66), however by 2010, children in the poorest economic quintile were 4 times more likely to be stunted than the richest (OR = 0.24; 95% CI:0.13,0.45; *p* < 0.001). The effect of maternal education on stunting prevalence continued over the 10 years, in both 2000 and 2010 children were twice as likely to be stunted if their mother had no education compared to university education (2000 OR = 0.45; 95% CI:0.28,0.73, *p* < 0.01,2010 OR =0.55; 95% CI:0.35,0.87, *p* < 0.05).

**Conclusion:**

Economic growth in Mongolia from 2000 to 2010 resulted in an increase in the effect of social determinants of child health; whilst focused policy improved access to immunisation. Children with less educated mothers and lower household incomes should be targeted in interventions to reduce health inequity.

## Background

Absolute poverty has long been highlighted as the enemy to adequate health across the world, however as the numbers living in poverty decrease [1], a new challenge to health is emerging; economic and educational inequality [2]. Unequal income distribution has a detrimental effect on health indicators even after adjusting for total income [3]. Evidence suggests that economic growth can exacerbate health inequities in LMICs [4] and as hugely populated countries, such as India and Nigeria, emerge from their low-income status, the suffering of the poor millions worsen [5–8]. For example, in India in 2005–06 infant mortality rate (IMR) among the poorest and richest wealth quintiles was 82 and 34 per 1000 births, respectively [8]. Similarly, in Nigeria in 2008, IMR was 87 and 219 per 1000 births in these groups [7]. Tackling inequality is also paramount in the fight to improve health throughout the world [6]. Interventions to improve health in vulnerable populations in LMICs have been proven to be more cost effective than mainstream approaches [9]. Analysis of the determinants of health and inequalities is important to identify vulnerable groups and to design and implement effective, targeted interventions policies to reduce health inequities [9–11].

There is scanty data from Central Asia and Eastern Europe on health inequalities where their historic socialist socio-political and economic policies still influence current policies. Mongolia is one such middle-income country that underwent the world’s fastest economic growth in the 2000s with foreign investment in the growth of its mining industry and it is predicted to continue growing, with Citigroup naming Mongolia as one of the 11 countries with most promising growth from 2010 to 2050 [12]. Trends for the Gini coefficient over the decade show an increase from 0.33 to 0.37, whereas the poverty rate remained static at 35% [12]. This shows that those who are already wealthier, benefit more from the economic growth, without reducing the overall poverty level. It is of interest to investigate how healthcare distribution changed in Mongolia, as an example of this region, where several countries are undergoing economic growth.

We chose indicators for child health and healthcare access during the period of economic growth. Birth to five-years of age carries a high chance of mortality and morbidity, and therefore indicators for this age group provide a measure of health inequities [13]. Furthermore, inequalities in this age group are significant because poor childhood health and development have detrimental implications on adult health, thus increasing population’s future health inequities [14]. Health Indicators, such as growth/nutrition and healthcare access, affect childhood morbidity and mortality; many socioeconomic and environmental factors such as maternal education, income and access to clean water have been shown to influence them [13, 15–20].

Therefore, this study used national representative data from Mongolian multiple indicator cluster surveys (MICS) to investigate the role of socioeconomic and environmental factors in child health, specifically growth and immunisation) during the period of economic growth 2000-2010.

## Methods

### Setting

Mongolia has much in common with the other Central Asian countries where in spite of low-middle income country status, education level is high with over 94% of males and females attend primary school [21]. Socio-political transition from a socialist system has led to huge changes since the 1990s in these countries.

In Mongolia, parallel to most other such countries, the changes led to a new health insurance funded system where the client requires a health insurance book to access healthcare with co-payments of varying levels depending on the service: 10% at the secondary care and 15% for tertiary care and outpatient drugs [22]. This replaced the socialist (Semashko) system in 1994, which provided universal health coverage free of charge. The new system has produced inequalities in healthcare access for adults and children, because the poor, especially the city migrants from the rural areas, are less likely to be registered with the city authorities to receive healthcare; the percentage of the population with health insurance decreased from 95.3% in 2000 to 82.6% in 2010 [23]. This results in them having to make out-of pocket (OPP) payments for healthcare, estimates for OPP rose sharply during the decade from 14.5% in 2000 to 41.4% in 2010 [12]. Policy included introduction of primary health care centres in urban areas from 2002 as part of a decentralised approach to managing healthcare; patients without health insurance could not access primary or secondary services without paying a fee [12].

Child health indicators collected in surveys, which encompass this unregistered population, demonstrate the reality of the situation [24]. Additionally, in Mongolia and numerous Central Asian countries, the nomadic lifestyle creates unique challenges to providing health care to the mobile population [25]. Mining and extreme weather have changed migration patterns and the health service needs to adapt to meet the needs of the growing urban [23] as well as difficult to access population.

### Data sources

This analysis uses the MICS data from 2000, 2005 and 2010. The methods for data collection are designed by UNICEF to produce comparable statistics within and between LMICs [26, 27]. The sample sizes and distribution are displayed in Table 1.

**Table 1:** Regional distribution of clusters, households and children sampled

	Clusters			Households			Children		
Regions	2000	2005	2010	2000	2005	2010	2000	2005	2010
West	56	41	84	875	571	750	1186	676	968
Khangai	69	60	84	1168	735	674	1598	843	820
Central	71	45	84	1060	540	728	1333	609	838
East	28	21	84	415	343	617	575	378	743
Ulaanbaatar	86	86	84	1237	779	606	1492	1041	745
Total	310	253	420	4755	2998	3375	6184	3547	3956

The total numbers of clusters, households and children sampled via the MICS surveys. Households sampled in the survey who had children under age 5 residing in them. Although the total number of households surveyed by MICS in each year was similar, the number of households with children reduced and the number of children under age 5 in those households also reduced leaving an overall reduction in the number of children surveyed in 2005 and 2010

### Measures

All indicators of child health used in this analysis are defined in Table 2. Growth was measured by both continuous (HAZ and WAZ) and categorical variables (stunted/non stunted and underweight/non-underweight status of children) [28]. Stunting is more important than underweight for measuring inequities [29] as it develops over a longer term and is a better indicator of nonacute factors affecting growth. Immunisation coverage has been used as an indicator of access to healthcare in previous studies exploring health inequity in Africa [30]. Immunisation in Mongolia was implemented through the Extended Programme for Immunisation (EPI) which was introduced in 1994 when the socialist regime ended. This programme was supported by GAVI (Gobal alliance for vaccines and immunsaitons) from 2001 with both financial and technical support [12]. Immunisations are provided by the primary health centres in the urban areas and by the district hospitals in rural areas. Parents are informed about necessary immunisations at child birth, most of which are in institutions in Mongolia. [31]

**Table 2:**
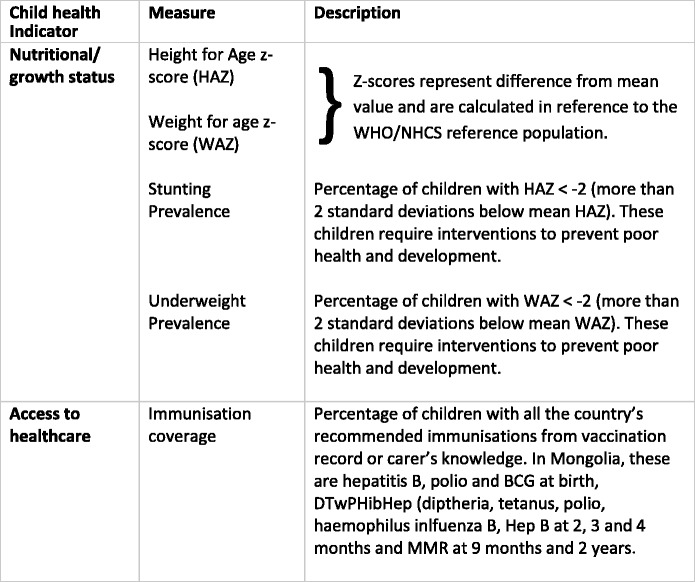
Child Health Indicators

Independent factors based upon evidence and available data were distributed across three levels; individual, household and community (Table 3). Economic status was measured by the wealth index, which was calculated by principal component analysis using housing type and materials, availability of electricity and household assets [32]. The households were divided into five wealth quintiles when data was collected through the MICS survey. Therefore, there are different numbers of children in each quintile through the years, depending on the percentage of children living in households within each quintile.

**Table 3:** Distribution (%) of variables

Variables		2000 (%)	2005 (%)	2010 (%)
	Total children	6184	3547	3956
Individual level factors				
Sex	Male	3148 (50.9)	1841(51.9)	1990 (50.3)
	Female	3032 (49.0)^a^	1706 (48.1)	1966 (49.7)
Age	0-11 months	1412(22.8)	774 (21.8)	819 (20.7)
	12-23 months	1256 (20.3)	723 (20.4)	914 (23.1)
	24-35 months	1192 (19.3)	714 (20.1)	859 (21.7)
	36-47 months	1120 (18.1)	672 (19.0)	713 (18.0)
	48-59 months	1204 (19.5)	664 (18.7)	651 (16.5)
Household level factors				
Socio-economic:	1	3418 (55.3)	2474 (69.7)	2561 (64.7)
Number of children	2	2446 (39.6)	963 (27.2)	1285 (32.5)
under age 5 in the home	3	312 (5.0)	110 (3.1)	104 (2.6)
	4	8 (0.1)	0 (0.0)	4 (0.1)
Maternal Education level	None	97 (1.6)	162 (4.6)	235 (5.9)
	Primary	1958 (31.7)	299 (8.4)	366 (9.3)
	Secondary	2064 (33.4)	1920 (54.1)	1857 (46.9)
	Vocational	648 (10.5)	252 (7.1)	258 (6.5)
	University	1417 (22.9)	914 (25.8)	1240 (31.3)
Household Wealth Index	Poorest	1424 (23.0)	813 (22.9)	1213 (30.7)
	Second	1273 (20.6)	842 (23.8)	874 (22.1)
	Middle	1199 (19.4)	686 (19.3)	706 (17.8)
	Fourth	1160 (18.8)	579 (16.3)	608 (15.4)
	Richest	1128 (18.2)	627 (17.7)	555 (14.0)
Environmental:				
Water source	Poor	2476 (40.0)	1156 (32.6)	1393 (35.2)
	Improved	3708 (60.0)	2391 (67.4)	2561 (64.7)*
Sanitation facility	Poor	4947 (80.0)	959 (27.0)	999 (25.3)
	Improved	1237 (20.0)	2588 (73.0)	2951 (74.6)*
Community level factors				
Place of Residence	Rural	3499 (56.6)	1707 (48.1)	2209 (55.8)
	Urban	2685 (43.3)	1840 (51.9)	1747 (44.2)
Region of Residence	West	1186 (19.2)	676 (19.1)	959 (24.2)
	Khangai	1598 (25.8)	843 (23.7)	787 (19.9)
	Central	1333 (21.6)	609 (17.2)	810 (20.5)
	East	575 (9.3)	378 (10.7)	709 (17.9)
	Ulaanbaatar	1492 (24.1)	1041 (29.3)	694 (17.5)

Improved drinking water sources: household connections, public standpipe, borehole, protected dug well, protected spring or rainwater collection. Improved sanitation facilities: connection to a public sewer, connection to a septic system, pour-flush latrine, simple pit latrine or ventilated improved pit latrine. ^a^Percentages do not equal 100 due to missing data

### Statistical analysis

MICS data have a hierarchical sample selection methodology, [26] [27] with children nested within households and households nested within communities. Observations, e.g. immunisation coverage, from those living in the same area are likely to be correlated because they share characteristics e.g. a local health clinic. Consequently, the standard assumption of the independence of observations in conventional regression models is violated and the model must be adjusted for the clustering of the data. [33] As a result, logistic regression models fitted for the categorical variables were adjusted for clustering using the generalised linear mixed model procedure [34]. The determining factors were all entered separately first (results not shown) and then variables found to be significant were entered into a multivariate model (Table 5) in order to calculate odds ratios for each independent variable. Because HAZ and WAZ were normally distributed, t-tests were used to test for significant change over time. The comparison across the years was carried out for each independent factor i.e. sex, age groups etc.

Regarding missing data, 4.8%, 5.8% and 6.0% of growth indicator data was missing for 2000,2005 and 2010 respectively, but no data on immunisations was missing. The distribution of the independent variables (age, sex, household wealth index etc) for those who had missing growth indicators was not significantly different from the rest of the sample.

## Results

### Health status measured through nutritional/growth indicators

#### HAZ and prevalence of stunted children

The mean HAZ score for Mongolian children fluctuated from 2000 to 2010 as the values improved from −1.12(SD1.53) in 2000 to −0.48(SD2.85) in 2005 but then declined slightly to −0.72(SD 1.39) in 2010(Table 4). This change was statistically significant (*p* < 0.001) from both 2000 to 2005, and 2005 to 2010 (Table 4). In contrast, the prevalence of stunted children decreased throughout the period: 25.5%, 20.5% and 13.3% were stunted in 2000, 2005 and 2010 respectively (Table 4).

**Table 4:** Exact Means of HAZ and WAZ and prevalence of stunted, underweight and immunised children

		Mean HAZ (±SD)				Mean WAZ (±SD)				
		2000	2005		2010	2000		2005	2010	
Sex	Male	−1.18 (1.50)	−0.55 (2.80)***		−0.74 (1.38)**	−0.65 (1.36)		−0.23 (1.30) ***	−0.11 (1.21)**	
	Female	−1.06 (1.55)	−0.41 (2.91)***		−0.69 (1.40)***	−0.54 (1.36)		−0.19 (1.36) ***	−0.07 (1.24)**	
Age	0-11 months	−0.55 (1.64)	0.17 (2.84) ***		−0.07 (1.42)*	−0.13 (1.48)		0.47 (1.37) ***	0.58 (1.30)	
	12-23 months	−1.43 (1.61)	−0.98 (2.47) ***		−1.00 (1.37)	−0.74 (1.40)		−0.31 (1.30) ***	−0.27 (1.27)	
	24-35 months	−1.14 (1.41)	−0.51 (2.82) ***		−0.78 (1.34)*	−0.69 (1.29)		−0.36 (1.21) ***	−0.23(1.12)*	
	36-47 months	−1.29 (1.34)	−0.57 (3.00) ***		−0.87 (1.32)*	−0.71 (1.25)		−0.51 (1.18) **	−0.28 (1.08)***	
	48-59 months	−1.20 (1.39)	−0.59 (2.02) ***		−0.90 (1.26)*	−0.79 (1.21)		−0.46 (1.31) ***	−0.32(1.02)*	
Number of children under age 5 in the home	1	−0.93 (1.46)	−0.41 (2.81) ***		−0.63 (1.33)**	−0.46 (1.34)		−0.16 (1.32) ***	−0.04(1.24)**	
	2	−1.32 (1.57)	−0.66 (2.83) ***		−0.86 (1.47)*	−0.73 (1.37)		−0.33 (1.33) ***	−0.17 (1.19)**	
	3	−1.61 (1.53)	−0.56 (3.75) **		−0.97 (1.59)	−1.01 (1.40)		−0.50 (1.33) **	−0.40 (1.22)	
	4	0.14 (1.66)	n/a		−3.91 (1.41)	−0.19 (0.69)		n/a	−1.41 (0.91)	
Maternal Education level	None	−1.59 (1.41)	−0.93 (2.84) *		−1.27 (1.22)	−1.10 (1.35)		−0.55 (1.29) **	−0.51 (1.19)	
	Primary	−1.42 (1.50)	−0.74 (2.64) ***		−1.04 (1.44)	−0.81 (1.38)		−0.46 (1.21) ***	−0.39 (1.15)	
	Secondary	−1.04 (1.49)	−0.63 (2.76) ***		−0.80 (1.38)*	−0.52 (1.35)		−0.31 (1.33) ***	−0.13 (1.22)***	
	Vocational	−1.13 (1.46)	−0.40 (2.75) ***		−0.65 (1.29)	−0.69 (1.30)		−0.10 (1.38) ***	−0.07 (1.19)	
	University	−0.78 (1.57)	−0.04 (3.08) ***		−0.40 (1.37)***	−0.32 (1.32)		0.09 (1.29) ***	−0.13 (1.23)	
Household economic status	Poorest	−1.45 (1.46)	−0.81 (2.61) ***		−1.04 (1.33)**	−0.82 (1.38)		−0.49 (1.21) ***	−0.33 (1.16)**	
	Second	−1.35 (1.52)	−0.83 (2.68) ***		−0.84 (1.47)	−0.74 (1.38)		−0.40 (1.25) ***	−0.15 (1.24)***	
	Middle	−1.17 (1.51)	−0.41 (2.92) ***		−0.65 (1.27)*	−0.67 (1.28)		−0.21 (1.33) ***	−0.03(1.23)*	
	Fourth	−0.95 (1.52)	−0.12 (3.05) ***		−0.51 (1.43)**	−0.47 (1.38)		0.04 (1.44) ***	−0.05 (1.23)	
	Richest	−0.55 (1.46)	−0.02 (3.01) ***		−0.11 (1.28)	−0.17 (1.44)		0.17 (1.35) ***	−0.31 (1.20)	
Water source	Poor	−1.41 (1.48)	−0.60 (2.75) ***		−0.85 (1.38)**	−0.82 (1.37)		−0.35 (1.23) ***	−0.17(1.21)***	
	Improved	−0.92 (1.52)	−0.43 (2.90) ***		−0.64 (1.39)**	−0.44 (1.36)		−0.15 (1.37) ***	−0.04 (1.23)**	
Sanitation facility	Poor	−1.25 (1.51)	−0.75 (2.57) ***		−1.01 (1.32)**	−0.69 (1.37)		−0.44 (1.25) ***	−0.30 (1.14)*	
	Improved	−0.57 (1.44)	−0.38 (2.94) *		−0.62 (1.40)***	−0.19 (1.27)		−0.13 (1.35)	−0.02 (1.24)**	
Place of Residence	Urban	−0.82 (1.53)	−0.30 (3.00) ***		−0.51 (1.40)**	−0.40 (1.31)		−0.05 (1.42) ***	0.08 (1.28)**	
	Rural	−1.35 (1.49)	−0.69 (2.67) ***		−0.88 (1.36)**	−0.74 (1.38)		−0.39 (1.20) ***	−0.23 (1.16)***	
Region of Residence	Ulaanbaatar	−0.66 (1.51)	0.04 (3.45) ***		−0.38 (1.46)***	−0.29 (1.26)		0.14 (1.55) ***	0.22 (1.39)	
	West	−1.40 (1.43)	−0.58 (3.07) ***		−1.08 (1.24)***	−0.75 (1.34)		−0.49 (1.16) ***	−0.33 (1.16)**	
	Khangai	−1.39 (1.53)	−0.82 (2.08) ***		−0.80 (1.44)	−0.77 (1.38)		−0.45 (1.17) ***	−0.14 (1.18)***	
	Central	−0.99 (1.52)	−0.66 (2.01) ***		−0.52 (1.37)	−0.41 (1.40)		−0.13 (1.21) ***	−0.05 (1.22)	
	East	−1.26 (1.51)	−0.74 (3.10) **		−0.68 (1.14)	−0.99 (1.32)		−0.27 (1.26)	−0.07 (1.14)*	
Total		−1.12 (1.53)	−0.48 (2.85)***		−0.72 (1.39)***	−0.59 (1.36)		−0.21(1.33)***	−0.09 (1.23)***	
		Stunting Prevalence			Underweight Prevalence			Full immunisation Prevalence		
		2000	2005	2010	2000	2005	2010	2000	2005	2010
Sex	Male	26.5	20.9	13.6	13.6	6.0	4.2	72.1	76.2	97.5
	Female	24.4	20.1	13.0	13.0	6.4	3.9	71.1	73.6	98.1
Age	0-11 months	16.6	11.1	5.2	9.1	2.8	2.3	33.4	82.6	97.9
	12-23 months	34.3	27.0	20.4	15.8	6.4	5.9	82.5	80.5	98.8
	24-35 months	23.0	19.2	13.4	14.1	9.0	4.5	83.5	74.6	98.1
	36-47 months	26.7	23.5	13.6	13.9	6.7	3.8	83.7	71.3	97.2
	48-59 months	28.0	22.7	13.5	14.3	6.5	3.5	82.3	63.9	94.9
Number of children under age 5 in the home	1	20.6	18.6	11.7	10.5	5.9	3.5	72.8	76.6	97.7
	2	30.8	24.1	15.9	16.4	6.9	4.9	70.2	70.7	98.1
	3	38.0	32.7	20.0	20.3	8.2	6.9	70.2	72.7	99.0
	4	0.0	n/a	n/a	100.0	n/a	n/a	75.0	n/a	n/a
Maternal Education level	None	38.7	32.1	24.6	19.4	13.0	9.8	63.9	73.5	97.9
	Primary	32.8	24.4	18.7	17.6	8.4	6.9	69.9	70.6	97.5
	Secondary	24.5	22.6	14.9	11.8	7.1	4.2	67.6	75.4	97.5
	Vocational	23.2	15.9	8.5	14.8	3.6	2.8	73.9	74.2	99.2
	University	16.8	14.1	8.3	8.3	3.2	2.1	79.3	75.8	98.1
Household economic status	Poorest	33.0	25.2	19.8	18.0	8.2	6.3	70.2	72.1	97.1
	Second	30.2	25.2	14.7	16.1	8.3	4.3	69.9	74.5	97.6
	Middle	27.2	19.8	10.2	12.7	5.2	3.4	70.5	77.1	97.9
	Fourth	21.8	15.0	9.8	11.8	4.3	2.3	73.1	77.5	98.5
	Richest	12.2	13.9	4.7	6.2	3.5	1.4	75.1	77.6	98.9
Water source	Poor	32.4	21.0	15.9	17.9	6.8	5.0	68.6	71.4	98.0
	Improved	20.8	20.2	11.9	10.2	5.9	3.6	73.7	76.6	97.7
Sanitation facility	Poor	28.7	24.1	19.0	15.0	6.4	5.9	70.8	70.6	97.1
	Improved	12.3	19.2	11.4	9.1	5.1	3.4	74.9	76.5	98.1
Place of Residence	Urban	18.9	18.4	10.4	9.5	5.4	2.8	72.6	73.4	98.2
	Rural	30.5	22.8	15.6	16.2	7.1	5.0	70.9	76.5	97.5
Region of Residence	Ulaanbaatar	15.3	18.3	9.6	7.3	5.0	2.8	80.2	72.0	99.0
	West	29.9	25.7	19.0	15.4	7.5	5.9	75.0	64.8	98.0
	Khangai	32.9	19.0	13.5	16.7	7.5	4.6	69.1	74.3	95.8
	Central	22.7	15.9	10.8	10.5	4.9	3.3	69.5	87.7	97.8
	East	28.2	27.8	11.9	21.4	6.3	3.0	54.4	81.7	98.7
Total		25.5	20.5	13.3	13.3	6.2	4.1	71.6	74.9	97.8

This table gives both the mean values for HAZ and WAZ and the prevalence (by percentage) of stunted, underweight and immunised children over the years by each health indicator used to measure equality. Statistical significance of change 2000 to 2005 (2005 values) or 2005 to 2010 (2010 values): *p < 0.05, **p < 0.01, ***p < 0.001

**Fig. 1:**
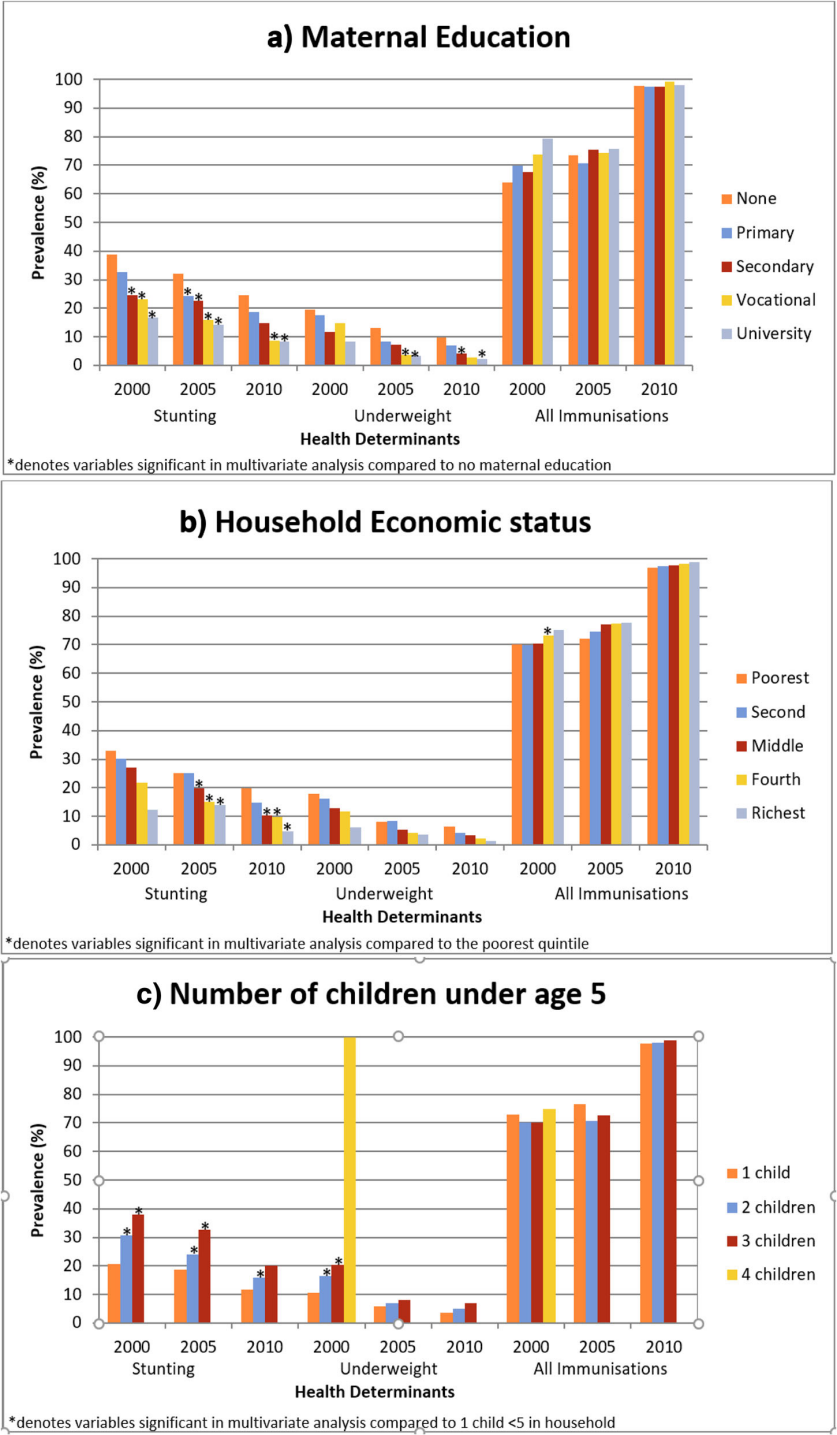
Distribution of stunting, underweight and immunisations across socio-economic household factors in 2000, 2005 and 2010. This figure depicts the prevalence of stunted, underweight and fully immunised children across the years 2000, 2005 and 2010. The colours represent the different determinants of health (**a**) maternal education, (**b**) household economic status and (**c**) total number of children under age 5 in the household. The * denotes the variables which were significant determinants of the outcome in multivariate analysis

The independent significant factors (from the multivariate analysis) affecting the stunting prevalence varied from 2000 to 2010 (Table 5). In 2000, sex, age, number of children <5 in the home, maternal education, sanitation facility and region of residence were all significant predictors of stunting. Female children were less likely to be stunted, OR = 0.87(95% CI0.77, 0.98) (Table 5). Compared to the children aged 0-11 months, children aged 12-23 months were the most likely to be stunted, OR = 2.83(95% CI 2.29, 3.51) (Table 5). The higher the number of children in the home, the more likely they were to be stunted, OR = 1.99 (95% CI 1.50, 2.65) for households with 3 children <5 years (Table 5, Fig. 1c). A higher level of maternal education reduced the prevalence of stunting, OR = 0.45(95% CI 0.28, 0.73) for children whose mother had a university education compared to those with no education (Table 5, Fig. 1a). An improved sanitation facility was protective against stunting, OR = 0.57(95% CI 0.76, 1.22). Lastly, living in the poorer Western, Khangai, or Eastern region increased the likelihood of stunting, OR = 1.59(95% CI 1.19, 2.13), 1.75 (95% CI 1.31, 2.32) and 1.48(95% CI 1.07, 2.04) respectively (Table 5). Household economic status was not a significant factor.

**Table 5:** Multivariate multilevel logistic mixed model regression estimates (odds ratios) of determinants of binary health indicators

		Stunting odds ratios (95% CI)			Underweight odds ratios (95% CI)		
		2000	2005	2010	2000	2005	2010
Sex	Male^a^				†	†	†
	Female	0.87 (0.77-0.98)*	0.95 (0.80-1.13)	0.97 (0.79-1.19)			
Age	0-11 months^a^						
	12-23 months	2.83 (2.29-3.51)***	2.94 (2.20-3.94)***	5.03 (3.47-7.29)***	1.90 (1.48-2.45)***	1.58 (1.02-2.46)*	1.66 (1.07-2.58)*
	24-35 months	1.55 (1.24-1.93)***	1.77 (1.31-2.40)***	2.88 (1.95-4.23)***	1.65 (1.27-2.13)***	2.07 (1.36-3.160**	1.36 (0.86-2.15)
	36-47 months	1.90 (1.52-2.37)***	2.33 (1.73-3.15)***	2.69 (1.80-4.01)***	1.60 (1.22-2.080**	1.63 (1.04-2.54)*	1.19 (0.73-1.94)
	48-59 months	2.06 (1.65-2.56)***	2.25 (1.66-3.05)***	2.79 (1.86-4.19)***	1.68 (1.30-2.18)***	1.59 (1.02-2.49)*	1.17 (0.71-1.94)
Number of children under age 5 in the home	1^a^						
	2	1.47 (1.29-1.69)***	1.30 (1.06-1.58)*	1.24 (1.01-1.57)*	1.26 (1.15-1.62)***	1.05 (0.79-1.41)	1.13 (0.83-1.53)
	3	1.99 (1.50-2.65)***	1.84 (1.14-2.96)*	1.55 (0.86-2.82)	1.69 (1.19-2.39)**	1.15 (0.57-2.31)	1.43 (0.67-3.07)
Maternal Education level	None^a^						
	Primary	0.73 (0.46-1.16)	0.62 (0.39-0.98)*	0.72 (0.46-1.13)	0.92 (0.52-1.63)	0.66 (0.36-1.21)	0.74 (0.41-1.35)
	Secondary	0.59 (0.37-0.94)*	0.60 (0.41-0.89)*	0.71 (0.49-1.04)	0.69 (0.39-1.22)	0.62 (0.38-1.04)	0.59 (0.36-0.98)*
	Vocational	0.53 (0.32-0.88)*	0.43 (0.25-0.72)**	0.47 (0.26-0.85)*	0.89 (0.49-1.64)	0.41 (0.19-0.87)*	0.53 (0.24-1.15)
	University	0.45 (0.28-0.73)**	0.45 (0.28-0.71)**	0.55 (0.35-0.87)*	0.57 (0.31-1.04)	0.44 (0.24-0.83)*	0.49 (0.27-0.92)*
Household economic status	Poorest^a^						
	Second	0.98 (0.81-1.18)	0.90 (0.68-1.19)	0.70 (0.48-1.00)	0.94 (0.74-1.19)	1.13 (0.76-1.67)	0.65 (0.31-1.40)
	Middle	1.03 (0.82-1.29)	0.64 (0.44-0.92)*	0.48 (0.31-0.75)**	0.82 (0.61-1.10)	0.84 (0.50-1.43)	0.69 (0.35-1.34)
	Fourth	0.90 (0.69-1.16)	0.49 (0.32-0.75)**	0.47 (0.29-0.76)**	0.95 (0.69-1.32)	0.80 (0.43-1.47)	0.79 (0.44-1.43)
	Richest	0.91 (0.49-1.66)	0.48 (0.30-0.77)**	0.24 (0.13-0.45)***	0.78 (0.36-1.67)	0.75 (0.39-1.45)	0.81(0.49-1.34)
Water source	Poor^a^				†	†	†
	Improved	0.99 (0.81-1.20)	1.23 (0.97-1.55)	1.0 (0.78-1.27)			
Sanitation facility	Poor^a^				†	†	†
	Improved	0.57 (0.33-0.97)*	1.04 (0.80-1.35)	1.06 (0.76-1.47)			
Place of Residence	Urban^a^						
	Rural	0.96 (0.76-1.22)	0.89 (0.64-1.26)	0.84 (0.61-1.16)	0.93 (0.68-1.28)	0.80 (0.53-1.22)	1.05 (0.70-1.59)
Region of Residence	Ulaanbaatar^a^						
	West	1.59 (1.19-2.13)**	1.07 (0.73-1.58)	1.39 (0.91-2.15)	2.55 (1.67-3.91)***	1.05 (0.66-1.67)	1.14 (0.66-1.98)
	Khangai	1.75 (1.31-2.32)***	0.73 (0.50-1.08)	0.94 (0.60-1.46)	1.31 (−.90-1.90)	1.10 (0.70-1.71)	0.99 (0.57-1.73)
	Central	1.28 (0.98-1.67)	0.66 (0.44-0.98)*	0.91 (0.58-1.42)	1.78 (1.20-2.63)**	0.87 (0.54-1.40)	0.93 (0.53-1.64)
	East	1.48 (1.07-2.04)*	1.23 (0.79-1.91)	0.94 (0.59-1.47)	1.76 (1.19-2.61)**	1.00 (0.59-1.69)	0.64 (0.46-1.51)
Log-Likelihood		−13,690.34	−8410.07	−18,777.09	−14,821.67	−9651.77	−20,591.32
Classification: % correct		76.9	80.5	87.1	86.9	93.8	96.0
Number of children		5927	336	3713	5927	3363	3716
		Immunisation odds ratios (95% CI)		2010			
		2000	2005				
Sex	Male^a^	†	†	†			
	Female						
Age	0-11 months^a^						
	12-23 months	2.29 (1.78-2.95)***	0.78 (0.57-1.07)	0.86 (0.53-1.42)			
	24-35 months	2.87 (2.21-3.73)***	0.52 (0.38-0.71)***	0.98 (0.60-1.60)			
	36-47 months	2.78 (2.13-3.64)***	0.41 (0.30-0.56)***	1.13 (0.67-1.85)			
	48-59 months	2.36 (1.82-3.06)***	0.25 (0.18-0.33)***	1.22 (0.74-2.01)			
Number of children under age 5 in the home	1^a^	†	†	†			
	2						
	3						
Maternal Education level	None^a^						
	Primary	1.18 (0.64-2.18)	0.72 (0.41-1.27)	1.05 (0.46-2.39)			
	Secondary	1.14 (0.61-2.11)	0.87 (0.53-1.44)	1.15 (0.58-2.32)			
	Vocational	1.24 (0.64-2.40)	0.83 (0.45-1.53)	0.89 (0.34-2.34)			
	University	1.55 (0.81-2.95)	0.86 (0.50-1.50)	1.18 (0.54-2.58)			
Household economic status	Poorest^a^						
	Second	1.02 (0.78-1.34)	1.12 (0.80-1.57)	0.92 (0.57-1.47)			
	Middle	1.31 (0.92-2.21)	1.39 (0.91-2.12)	0.87 (0.50-1.51)			
	Fourth	1.50 (1.03-2.20)*	1.47 (0.91-2.36)	0.77 (0.41-1.45)			
	Richest	1.46 (0.89-2.40)	1.48 (0.91-1.93)	0.69 (0.33-1.45)			
Water source	Poor^a^						
	Improved	1.44 (1.08-1.90)*	1.25 (0.94-1.65)	1.18 (0.82-1.68)			
Sanitation facility	Poor^a^	†	†	†			
	Improved						
Place of Residence	Urban^a^						
	Rural	3.16 (1.71-5.82)***	1.68 (0.95-2.97)	0.96 (0.62-1.48)			
Region of Residence	Ulaanbaatar^a^						
	West	0.37 (0.16-0.82)*	0.61 (0.30-1.22)	1.07 (0.59-1.95)			
	Khangai	0.32 (0.15-0.71)**	1.09 (0.57-2.07)	1.54 (0.87-2.74)			
	Central	0.24 (0.12-0.50)***	2.64 (1.33-5.25)**	1.14 (0.63-2.07)			
	East	0.08 (0.03-0.20)***	1.57 (0.68-3.61)	0.94 (0.50-1.77)			
Log-Likelihood		−15,972.66	−8606.55	−22,040.53			
Classification: % correct		88.0	84.4	97.8%			
Number of children		6184	3547	3953			

Statistical significance of effect on outcome in multivariate analysis: *p < 0.05, **p < 0.01, ***p < 0.001. The model was adjusted for the cluster effect

†represents missing odds ratios are for variables not significant in bivariate analysis so not included in the multivariate model. Prevalence values for each outcome can be found in Table 4

^a^denotes the reference category

In 2005, the significant factors contributing to stunting were similar; however, sex, sanitation facility and region of residence were no longer significant in the multivariate analysis, showing their effect was overshadowed by the social determinants; maternal education and economic status. (Table 5). Children of weaning age, 11-24 months, were still the most likely to be stunted, OR = 2.94(95% CI 2.20, 3.94) compared to children aged 0-11 months. The trends that increasing number of children in the home and reduced maternal education increased likelihood of stunting were still present in 2005, OR = 1.99(95% CI 1.50, 2.65) and 0.45(95% CI 0.28, 0.73) respectively (Table 5, Fig. 1a and c), and a higher household economic status was protective against stunting, OR = 0.48(95% CI 0.30, 0.77) for children in the richest quintile compared to poorest (Table 5, Fig. 1b).

In 2010, the significant factors contributing to stunting prevalence were the same as in 2005 but ORs indicated increased effect of these factors (Table 5): higher maternal education was more protective than in 2005 (OR = 0.55 (95% CI 0.35,0.87) for children whose mothers had a university education) (Table 5, Fig. 1a), and higher household economic status was more protective against stunting (OR = 0.24 (95% CI 0.13,0.45) for children in richest quintile (Table 5, Fig. 1b).

### WAZ and prevalence of underweight children

The mean WAZ for children in Mongolia increased from 2000 to 2010: the values significantly increased from −0.59(SD 1.36) in 2000 to −0.21(SD 1.33) and −0.09(SD 1.23) in 2005 and 2010, respectively (Table 4). The prevalence of underweight children followed this trend and decreased from 2000 to 2005 and to 2010 at 13.3%, 6.2% and 4.1% respectively (Table 4).

During the regression analysis, the independent significant factors varied over the decade similarly to those affecting stunting prevalence. In 2000, age, number of children under 5 in the home and region of residence all significantly affected the prevalence of underweight children. For age, as with stunting, children aged 12-23 months were the most likely to be underweight (OR = 1.90 (95% CI 1.48, 2.45) Table 5). Also in 2000, the likelihood of underweight children increased with the number of children under-5 in the home, OR = 1.26(95% CI 1.15, 1.62) for 2 children and OR = 1.69(95% CI 1.19, 2.39) for 3 children (Table 5, Fig. 1c). The region of residence had an effect as children living in the Western, Central and Eastern regions were all significantly more likely to be underweight than those in Ulaanbaatar (OR = 1.55 (95% CI 1.67,3.91), 1.78 (95% CI 1.20,2.63) and 1.76 (95% CI 1.19,2.61) respectively, Table 5). Household economic status and mothers’ education were not significant factors in 2000.

In 2005, age was still a significant factor contributing to underweight status and the other significant factor was maternal education (Table 5, Fig. 1a). Children aged 12-23 were more likely to be underweight, OR = 2.07 (95% CI 1.36, 3.16) (Table 5). A higher level of maternal education was protective against underweight children, OR = 0.44 (95% CI 0.24, 0.83) for mothers with a university education (Table 5).

In 2010, the same factors as 2005 were still significantly contributing to the underweight status of children; child’s age and level of maternal education (Table 5). Children aged 12-23 compared to those aged 0-11 months were still more likely to be underweight, OR = 1.66 (95% CI 1.07, 2.58) and children whose mother had a university education were somewhat less likely to be underweight, OR = 0.49 (95% CI 0.27, 0.92) (Table 5, Fig. 1a). There were less independent significant factors overall from 2000 to 2010;.. however, mothers’ education became significant from 2000 to 2005 and its related OR increased in 2010.

Overall, these results have shown a reduction in the effect of environmental determinants of health, such as region of residence, across the decade. An important improvement is the lack of significance of rural and urban divide and housing sanitation. However, the effect ofmaternal education and household economic status on child nutritional status, has increased (Table 5).

### Access to healthcare measured through immunisation coverage

Over the 10 year period, the proportion of fully immunised children increased, the values were 71.6% in 2000, 74.9% in 2005 and 97.8% in 2010 (Table 4).

The significant factors contributing to the immunisation rate varied through the years. In 2000, age, living in a household with a water source, rural/urban place of residence and region of residence were statistically significant (Table 5). All children over 12 months were more likely to have full immunisation coverage (Table 5), and the presence of a water source in the home was associated with a higher likelihood of full immunisations (OR = 1.44 (95% CI 1.08, 1.90)). In all other regions, children were less likely to be fully immunised than those living in Ulaanbaatar (Tables 4 and 5) although in the rural areas children were more likely to be fully immunised (OR = 3.16 (95% CI 1.71, 5.82)).

In 2005, only age and region of residence were significant contributors to the immunisation rate (Table 5). The trend in age however had reversed from 2000 as children over 12 months were less likely to have full immunisation coverage (Table 5). The older the child the less likely they were to be fully immunised (Table 5). This may be explained by the introduction of the Haemophilus Influenza vaccine between 2000 and 2005 which meant many older children were not vaccinated if they were born before it was introduced. [35] Only two regions of residence had significantly reduced immunisation coverage, the West region, and UB (Table 4).

In 2010, no variables had a significant effect on immunisation rate. Overall, there was a huge progress in immunisation coverage over the decade accompanied by a lack of significant variables in the multiple regression analysis (Table 5).

## Discussion

This paper is the first to identify sources of child health inequity in former Soviet/Socialist States and opportunistically examines changes in health inequality over a period of dramatic economic growth, revealing a case for both celebration and concern. We found that in the Mongolia, despite the improved GDP across the decade, the effect of social determinants on child health increased from 2005 to 2010. Significant improvements in some household conditions (sanitation) and mother’s education could not mask the dramatic effects of inequalities as measured by household wealth and maternal education in terms of child health outcomes. Stunting and HAZ rates, which were our best indicator for chronic nutritional deficiency and health improvement, as well as malnutrition and WAZ rates improved for the total population across the decade of 2000-2010, Immunisation, as an indicator of healthcare use, improved dramatically as policy and funding focus increased coverage nationwide in 2010.

This study was limited by the quality of the MICS data on which it was based. For example, an attempt was made to analyse disease burden; which is measured through MICS by asking specifically about respiratory and diarrhoeal illness only, alongside the other measures of health considered. However, scrutiny of raw, analysed data and questionnaires revealed that the data was problematic since if the child was confirmed to have diarrhoea in the 2 weeks preceding the questionnaire, they were not asked about respiratory symptoms and vice a versa thus making disease data unreliable in our MICS surveys as the variables were not collected independently. Immunisation coverage in MICS surveys is limited because it does not account for the fact that young children may not yet have reached the age to have all their recommended immunisations and therefore this is also unaccounted for in our results [36]. Another limitation of the MICS data was the lack of some maternal data, such as age and marital status for children > 1-year of age which prevented adjustments for these important factors. Multiple other studies have shown that maternal characteristics affect child health outcomes [37, 38].

The above limitations on reporting disease, immunisations and maternal factors indicate that researchers using MICS data from all countries should exercise caution when comparing MICS results between years and countries. They should take care to understand how the data was collected and defined during each data collection round, rather than automatically assume all survey data have the same standard and method of collection. Those designing MICS surveys need to attempt to reduce disparities in data collection. Nevertheless, from the information available on the methods of MICS surveys the data chosen and presented in our study is coherent and comparable.

Study strengths were the large representative national database with small amount of missing data, and analysis of the missing data showing no correlation with the variables of interest. Also, the use of MICS data is important as in comparison, official government data collection of health indicators in Mongolia can be considered less accurate, as an example due to the exclusion of a large proportion of the population who are unregistered with the health services [39]. For example, the under-5 mortality rate estimated by MICS is 45 per 1000 live births; 20/1000 higher than that of the state health statistics department that quoted 25 per 1000 live births [40, 41].

Our results are important given that inequity in child health was recognised as a significant barrier to achieving the Millennium Development Goals [29] (and now the Sustainable Development Goals). Although GDPs and health outcomes in most LMIC are improving, this masks an exacerbating divide between poorest, which include the socially vulnerable groups and those most benefitting from economic growth as demonstrated through the inverse care law [42]. Data from our region of Central Asia and Eastern Europe are scarce, but examples are documented for large populations such as Nigeria and India [6, 7]. Below we discuss the importance of the trends in health outcomes and inequalities stemming from our results.

### Trends in health outcomes

Overall, results confirm an improvement in nutritional/growth status and immunisation coverage for Mongolian children <5 years from 2000 to 2010, which follows the trend in other Central Asian countries [43–45]. However, in spite of its much higher economic growth rate in the decade, Mongolia had only a marginally higher percentage improvement in prevalence of poor nutrition over this time, still not catching-up with the other comparable countries of the region. For example, according to MICS data in 2000, Mongolia’s prevalence of stunting (25.5%) and underweight (13.3%) was worse than in other transitioning Central Asian countries; values from 1999 in Kyrgyzstan were 24.8% and 11.0% respectively and in Kazakhstan were 9.8% and 4.6% [46]. This improved to 13.1% stunted and 3.7% underweight in Kazakhstan, 12.9% and 2.8% in Kyrgyzstan compared to 13.3% and 4.1% in Mongolia [5].

Although overall stunting prevalence improved across the country from 2000 to 2005, it significantly deteriorated in Ulaanbaatar in 2005. The same pattern was seen for immunisation. This finding may be due to the huge increase in peri-urban populations in the city, where living conditions are poor, directly affecting children’s diets and indirectly affecting their psychosocial environment as well as the unregistered migrants having reduced access to healthcare [24]. The decrease in health outcomes for the capital city was targeted in health policy and the decline reversed by 2010, including government funding for the WHO ‘s Extended Programme for Immunisation in 2002 [46] There was also introduction of the ‘Reaching Every District (RED)’ strategy in Mongolia in 2008 [47] which was supported by the WHO and UNICEF and emphasised the need for improvements in child health throughout the country, focussing on the underserved unregistered new migrants in cities. RED, as well as NGO and other internationally funded new projects increased funding in child health [48]. The improved trends in 2010 data clearly demonstration how policy focus and funding can improve health service utilisation and outcomes.

### Trends in inequalities

Overall improvements or deterioration of indicators are not a marker of the status of inequalities in health or access to services, since mean rates can mask widening of gaps between/within defined populations such as by sex or socio-economic status. Across the MICS surveys, in Mongolia, gender was not a significant determinant of child health, in contrast to other Asian countrie [48]. This is an important observation possibly due to the reverse gender role in Mongolia, where women are better educated and more likely to have professional careers compared to men [49].

Our results identified that the social determinants of health have the greatest effect on stunting prevalence, an indicator of chronic nutritional deficiency and recommended by the WHO for measuring health equity [50]. Bivariate analysis demonstrated that all socioeconomic and environmental factors significantly affected stunting prevalence in all years analysed (data not shown), and although environmental factors were no longer significant in the multivariate model, economic status and the related indicator, maternal education, were independent mediators in both 2005 and 2010. Thus, Mongolia’s high female education rate could not mask the powerful cumulative effects that inequalities in maternal educational attainment has on chronic child health indicators such as stunting. Maternal education and economic status have been shown to be significant, independent determinants of nutrition/growth in other cross-national studies [51–55].

As household economic status from 2000 to 2010 significantly affected prevalence of stunting, it suggests that the economic growth in this time period was not universal and had a negative effect on the equity of children’s nutritional/growth status. This finding is reflected in other countries where economic growth has not necessarily resulted in a decline in socio-economic health inequities [14]. Marmot et al. demonstrate that even in a high-income country (HIC), economic growth does not automatically result in equitable improvements in health due to inequitable distribution of wealth and a recent report indicates that in the UK child inequalities have grown in the poorest group [14]. The example of Mongolia demonstrates that this principle applies to LMICs in former Soviet/Socialist countries as well as HICs.

The prevalence of poor nutrition was highest in the 12-23 months age group, consistent with research in other countries [56]. However, the low prevalence of malnourished children aged 0-11 months contradicts the global trends, which demonstrate WAZ to be lowest until 9 months of age [56]. These findings could be attributed to Mongolia having a high rate of prolonged breast-feeding [57] which is likely to reduce the chance of malnutrition in children aged 0-11 months. However, poor weaning strategies and diet [58, 59] are likely to be responsible for the increase in prevalence of malnutrition in older children. MICS in Mongolia (2000, 2005 and 2010) did not provide data on child-feeding practices to test this hypothesis.

Significantly, over the same 10 years, there is an apparent reduction in the effect of location, both regionally and urban vs rural, for *all* child health indicators measured, which is interesting given the high internal migration rate into Ulaanbaatar. The reduction in inequality between Ulaanbaatar and the rest of the country may be explained by the high numbers of unregistered internal migrants moving into the city who are not eligible to access the healthcare they require [23]. Therefore, the reduction in regional inequality may be explained by worsening outcomes in Ulaanbaatar rather as well as improvement in other regions, as shown by the significant reduction in immunisation rate in Ulaanbaatar from 2000 to 2005 (Table 4).

In spite of this, in 2010, the lack of significant disparity in immunisation coverage by maternal education and household economic status (present between 2000 and 2005) is unusual compared to other countries, in which both factors increase the likelihood of a child receiving all their immunisations [60–62]. These studies show that maternal education is often the most important factor. Therefore, Mongolia’s high female literacy rate [63], the increased government funding for immunisation since 1999 [64] and a tradition of high immunisation since the Soviet era, may be partly responsible for the reduced inequalities in access to healthcare, at least in terms of immunisation. Furthermore, we question the value of immunisation as a measure of access to healthcare because it could be argued that immunisation is easy to implement in an evidence-based, vertical programme with high coverage and therefore may not reflect all other healthcare access in the case of Mongolia. Future studies on determinants of health should also attempt to investigate other measures of healthcare access.

## Conclusion

This is an important study examining the effect of economic growth on healthcare in a LMIC where much funding for aid work has been reduced in the last few years. Increasing GDP is demonstrated not to be correlated with equal benefit for the most vulnerable and this poses an ethical question for the Mongolian Government to impose taxation and development policies which will benefit the poor as well as the newly rich. Such policies should enable the continuing economic growth without widening inequity in health. Lessons are pertinent to LMIC, but particularly other Central Asian and East European countries with similar healthcare systems in transition from previous socialist policy. Important issues are also highlighted about the methodology and use of MICS data.

## Abbreviations

CI: Confidence interval
GDP: Gross Domestic Product
HAZ: Height for Age z-score
HIC: high income country
IMR: Infant mortality rate
LMIC: lower middle income country
MICS: Multiple Indicator Cluster Survey
NGO: Non-governmental organisation
OR: odds ratio
RED: Reaching Every District Strategy
UNICEF: United Nations Children’s Fund
WAZ: Weight for Age z-score
WHO: World Health Organisation
