# Correction to: Social and environmental determinants of child health in Mongolia across years of rapid economic growth: 2000–2010

**DOI:** 10.1186/s12939-018-0747-7

**Published:** 2018-03-22

**Authors:** Nehal Joshi, Bolormaa Bolorhon, Indermohan Narula, Shihua Zhu, Semira Manaseki-Holland

**Affiliations:** 1 Medicine, University of Birmingham, Birmingham, UK; 2 Medical Student, Health Sciences University of Mongolia, Ulaanbaatar, Mongolia; 3 Team Leader, Global Fund LFA, Ulaanbaatar, Mongolia; 4 Research Fellow in Health Economics and Mathematical Modelling, Public Health, Epidemiology and Biostatistics, School of Health and Population Sciences, College of Medical and Dental Sciences, University of Birmingham, Birmingham, UK; 5 Clinical Senior Lecturer, Department of Public Health, Epidemiology and Biostatistics, School of Health and Population Sciences, College of Medical and Dental Sciences, University of Birmingham, Birmingham, UK

## Correction

Unfortunately, after publication of this article [1], it was noticed that an error during the production process resulted in an incorrect author name. The author Semira Manaseki-Holland is incorrectly displayed as Semira Manaseki-Hollan. The full, corrected author list can be seen above.
